# Movement of an imperiled esocid fish in an agricultural drain

**DOI:** 10.1186/s40462-023-00420-2

**Published:** 2023-12-13

**Authors:** Benjamin J. Zdasiuk, Marie-Josée Fortin, Julia E. Colm, D. Andrew R. Drake, Nicholas E. Mandrak

**Affiliations:** 1 Department of Ecology and Evolutionary Biology, University of Toronto, 25 Willcocks St., Toronto, ON M5S 3B2 Canada; 2 Great Lakes Laboratory for Fisheries and Aquatic Sciences, Fisheries and Oceans Canada, 867 Lakeshore Road, Burlington, ON L7S 1A1 Canada; 3 Department of Biological Sciences, University of Toronto Scarborough, 1265 Military Trail, Toronto, ON M1C 1A4 Canada; 4 Present Address: Simon Fraser University Biology, 8888 University Dr W, Burnaby, BC V5A 1S6 Canada

**Keywords:** Agricultural drains, Fish condition, Grass Pickerel (*Esox americanus vermiculatus*), Monitoring, Passive integrated transponder (PIT) tags, Species at risk

## Abstract

Animal movement is increasingly affected by human alterations to habitat and climate change. In wetland systems, widespread hydrologic alterations from agriculture have changed the shape, function, and stability of shallow streams and wetland habitats. These changes in habitat quality and quantity may be especially consequential for freshwater fishes such as Grass Pickerel (*Esox americanus vermiculatus*), a small predatory fish found in disjunct populations across southern Ontario and listed as Special Concern under Canada’s *Species at Risk Act*. To characterize Grass Pickerel movement response to stream-channel alterations, Fisheries and Oceans Canada implemented a tracking study to monitor the movements of a Grass Pickerel population in an agricultural drain on the Niagara Peninsula (Ontario, Canada). From 2009 to 2013, 2007 Grass Pickerel were tagged and tracked in the 37.3 km^2^ Beaver Creek watershed using a combination of mark-recapture surveys and eight fully automated passive integrated transponder tag antennas. Most individuals moved within 500 m (i.e., stationary fish) while 16% of the fish moved > 500 m (i.e., mobile fish), with a maximum median movement distance of 1.89 km and a maximum movement distance of 13.5 km (a long-tail distribution). Most movements occurred near the largest confluence where only a few were long-distance upstream or downstream movements. Mobile fish were larger than their stationary counterparts. Grass Pickerel in sites with higher abundance had more mobile fish, implying potential density dependence. Our results highlight that, while a long-distance dispersal ability exists in extant Grass Pickerel populations, the current conditions of riverscapes may prevent these dispersals from occurring. For declining Grass Pickerel populations, limitations to their movement ecology may substantially increase the likelihood of local extirpations.

**Supplementary Information:**

The online version contains supplementary material available at 10.1186/s40462-023-00420-2.

## Introduction

Abiotic conditions of streams and wetlands are changing globally. Aquatic systems face both direct anthropogenic disturbances, such as alterations to stream channels, and indirect effects of climate change, such as hydrologic changes due to variable precipitation patterns and increasing water temperatures. This can alter the range of physical conditions experienced by aquatic organisms and impair important ecological functions [19, 35].

Agricultural drains are a common altered aquatic system. Agricultural drains are artificial channels used to move water on and off arable land and a globally widespread feature of working agricultural landscapes [36]. To facilitate water transport to and from agricultural fields, natural waterways are frequently channelized, embedded, or diverted, changing both the timing and flow path of water moving through a riverscape [27]. In areas with intensive agricultural land use (e.g., central North America), the majority of catchment basin areas may drain to these artificial channels before reaching larger river systems [2]. Further, agricultural drain construction often reclaims or dries natural wetlands, contributing to the global loss of temperate wetland areas [42]. However, the inherent design characteristics of agricultural drains (shallow bank slopes, generally low flow, and comparatively warm water temperatures) may mirror pre-existing wetland habitats and serve as an alternate habitat type or even a biodiversity hotspot in working landscapes [36, 54]. Despite calls to manage agricultural drains as multiple-use systems (by providing drainage, fish habitat, and nutrient sinks,[75], there is a lack of knowledge of freshwater fish ecology in drains compared to larger waterways or naturally occurring wetlands [2].

As one of several critical ecological functions, fish movement may be impacted in agricultural drains, where decreases in total habitat area and flow volume can increase the likelihood and frequency of fragmentation events [79]. Stream-channel modifications and dredging activities risk fragmenting and isolating stream fish populations by linearizing and homogenizing fish habitats, which may impede passage during stressful environmental conditions [63]. These risks go against current conservation priorities to maintain or improve fish movement ecology and connectivity across metapopulations and diverse habitat types [56, 65]. Hence, to protect ecological function and fish biodiversity in agricultural drains, knowledge of movement extent, connectivity between population subgroups, and recolonization following disturbance events may be useful.

Over the last 200 years in southern Ontario, agricultural development and other land-use changes have driven a 68% or greater loss in total wetland area [61]. This widespread decline in wetland area across the region coincides with a high native richness of freshwater fishes, including imperiled species [7, 22]. Changes in the shape and extent of wetland habitat area experienced across the region may lead to mismatches between historical movement strategies (i.e., opportunistic movement during flood seasons, location of refugia during drought) and suitability of contemporary habitats. Prior work has shown freshwater fish communities in southern Ontario agricultural drains to be relatively representative of natural waterways and resilient to disturbances from maintenance activities [80, 84]. Yet, the movement characteristics of fishes in southern Ontario agricultural drains, and especially of threatened species, remain understudied [55].

For at-risk species—those with small effective population sizes or reduced allelic diversity across their populations—movement and connectivity are critical for maintaining gene flow and, in extreme cases, preventing mutational meltdown [58]. Despite the conservation needs to assess connectivity and movement in agricultural drainages, accurate fish movement and connectivity data are difficult to obtain due to the high cost of tagging and tracking fishes, as well as the computational demands for analyzing tracking data [3].

Here, we use fish movement of an at-risk species, Grass Pickerel (*Esox americanus vermiculatus*), in a southern Ontario agricultural drain as a case study for regions experiencing similar land-use change and freshwater biodiversity concerns. Grass Pickerel is listed as a species of Special Concern [13]. Grass Pickerel is a relevant fish species for studying movement in the aquatic-anthropogenic agricultural drains of southern Ontario [17]. It is a small (< 33 cm total length), predatory esocid, generally found in wetlands, low-order streams, and nearshore areas of lakes and rivers [16]. Some of the stream reaches with the highest recorded local abundances of Grass Pickerel are agricultural drains that mirror the shallow slopes, vegetated channels with ample floodplain habitat, and high-conductivity (i.e., clay-rich) substrate characteristics of naturally occurring wetland and stream Grass Pickerel habitat [9]. Its Canadian range consists of four disjunct populations that likely originated from a single Pleistocene refugium, but are now geographically and genetically distinct, with contemporary gene flow between populations unlikely [50, 51]. Further, a recent analysis of Niagara Peninsula (Ontario) subpopulations suggests that even geographically close subpopulations (< 30 river km) have limited gene flow, perhaps due to habitat barriers to functional movement [50].

Movement tendencies of Grass Pickerel are not well described in the literature. Kleinert and Mraz [43] observed Grass Pickerel moving towards a flooded slough forest in the spring for spawning in a Wisconsin lake; however, given the naturally dynamic (i.e., flashy in the winter and spring, prone to drying in the summer) stream systems that it more typically inhabits, movement ability may have ramifications for locating optimal habitat conditions, fitness, and survival. For other predatory fishes, including Northern Pike (*Esox Lucius*), Walleye (*Sander vitreus*), and Spotted Gar (*Lepisosteus oculatus*), migratory, resident, and intermittent movement strategies have all been documented around wetland habitat [31, 52, 53, 71, 78]. While parallels may exist between the movement characteristics of Northern Pike, Walleye, and Grass Pickerel, Grass Pickerel is far less likely to be found in deeper, pelagic habitat that larger-bodied predators (such as Northern Pike and Walleye) occupy. Conversely, Grass Pickerel is likely able to tolerate warmer water temperatures and lower oxygen concentration conditions than many larger piscivores [9]. Crossman [16] suggested that Grass Pickerel may move both seasonally and in response to fragmentation risk as streams or wetlands dry. The ecological needs for movement to maintain gene flow, locate adequate forage and habitat, and seek refuge from disturbance (e.g., droughts) is likely heightened for Grass Pickerel in contemporary agricultural drainages, where habitat alterations like dredging may increase habitat heterogeneity or create patchier habitat networks. It is in an agricultural drain of southern Ontario that we describe the movement characteristics of a Grass Pickerel population and whether movement is linked to temporal, environmental (habitat characteristics), or individual (condition, age, survival) attributes.

The objectives of this study were to: (1) determine the prevalence and distances traveled by Grass Pickerel in an agricultural drainage subject to multiple disturbances (dredging and drought); (2) test whether movement is linked to length or physiological condition differences in Grass Pickerel; and, (3) test whether movements are related to habitat and temporal conditions. To do so, we used passive integrated transponder (PIT) tags to measure an approximate proportion of the population moving—here opposing stationary fish (moving < 500 m) and mobile fish (moving > 500 m)—and displacements linked to each fish. To assess length and physiological condition differences, we used measurements of recaptured fish to test if there were condition or length differences between stationary and mobile population proportions. Lastly, we evaluated the relationship between movement and habitat by relating immigration to a site, emigration from a site, and distribution of stationary and mobile population proportions to habitat variables.

## Methods

To monitor Grass Pickerel movements and potentially relevant habitat conditions, a combined movement-tracking and habitat-survey program was implemented in an agricultural drain in southern Ontario in collaboration with the Municipality of Fort Erie, Fisheries and Oceans Canada (DFO), Ontario Ministry of Natural Resources and Forestry, and other local stakeholders. Monitoring included frequent characterizations of the fish community, substrate, and vegetation conditions at several sites throughout the watershed, and extensive tagging of Grass Pickerel with PIT tags. These tags were tracked with eight stationary antennas placed throughout the watershed from 2009 to 2013. During the study period, part of the study area was dredged in the fall of 2011, and a drought affected the watershed in the summer of 2012. These circumstances provided an opportunity to study Grass Pickerel movement in a before-after-control-impact (BACI) design [32].

### Study watershed

**Fig. 1:**
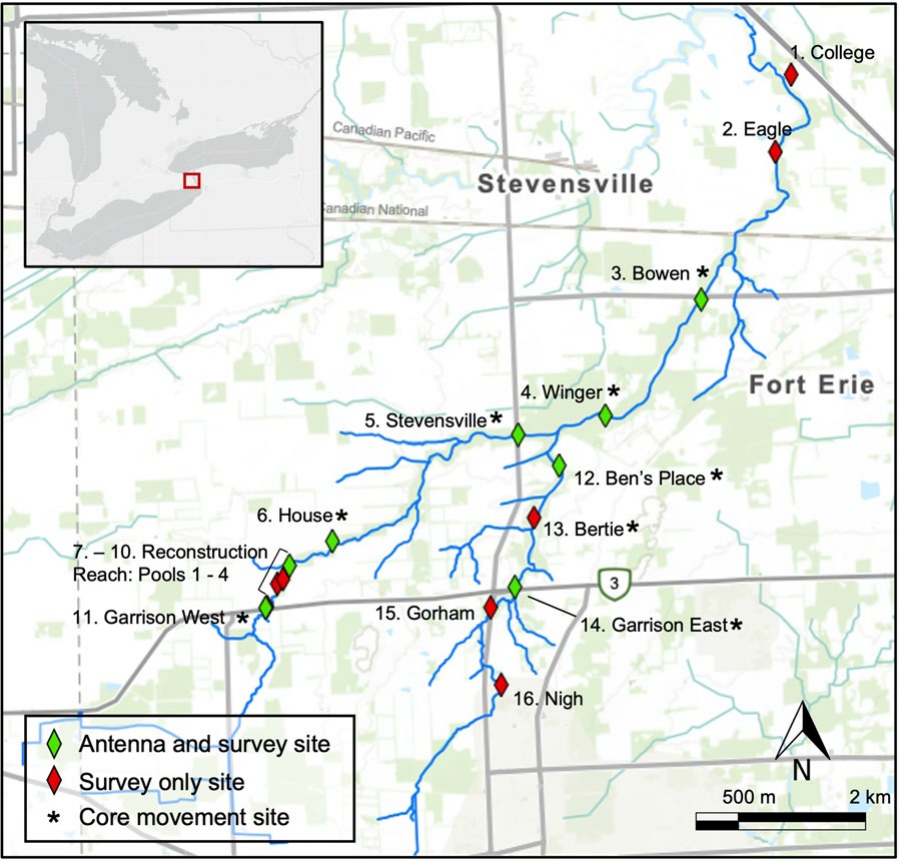
Location of antennas, core movement, and survey sites in Beaver Creek (Ontario) from 2009–2013 (see Table 1 for more details)

The Beaver Creek watershed (Fig. 1) is a low-gradient, 37.3 km^2^ watershed in a rural area of Fort Erie, Ontario, that drains into Black Creek, a tributary to the Niagara River. The creek is characterized by a 16.8 km north-easterly primary channel with a 7.6 km long south tributary [76]. From the confluence of the southern tributary and primary channel downstream to the confluence of Black Creek, the creek is designated a municipal drain and subject to municipal drain maintenance ordinances from the Municipality of Fort Erie. As such, the primary channel morphology shows signs of alteration including channel realignments, removal of meanders, and artificial entrenchment. The southern tributary is comparatively natural and does not show the same geomorphic alterations [76]. The substrate in the system is predominately clay, with short reaches of silt, gravel, or cobble on clay or silt on bedrock [76]. Channel entrenchment and riparian conditions vary throughout the watershed with sinuous pool-riffle sequences, shallow vegetated floodplains, and highly entrenched channels all represented in the watershed [76].

**Table 1:** Description of sampling locations, Grass Pickerel tagged, and Grass Pickerel detected in Beaver Creek, Ontario, 2009–2013

Site	Creek branch	Coordinates (latitude, longitude)	Upstream area (km^2^)	Antenna	Grass pickerel tagged	Grass pickerel detected (antenna)	Grass pickerel recaptured (survey)
1. College Rd	Mainstem	42.95555, − 79.01482	37.33	No	22		1
2. Eagle St		42.94767, − 79.01727	32.98	No	182		9
3. Bowen Rd		42.93273, − 79.02808	29.90	Yes	427	284	57
4. Winger Rd		42.92107, − 79.0418	24.92	Yes	141	74	5
5. Stevensville Rd	West branch	42.91933, -79.05402	13.54	Yes	289	340	73
6. House Rd		42.9108, -79.0516	9.88	Yes	139	61	16
7. Reconstruction Pool 1		42.9070, -79.08531	9.35	No	0		0
8.. Reconstruction Pool 2		42.9065, -79.08628	9.35	Yes	1	3	0
9. Reconstruction Pool 3		42.90513, -79.08723	9.35	No	3		0
10. Reconstruction Pool 4		42.90466, -79.08804	9.35	No	1		0
11. Garrison Rd. West		42.90223, -79.08973	9.35	Yes	47	33	6
12. Ben's Place	South branch	42.91612, -79.04842	11.38	Yes	33	64	1
13. Bertie Rd		42.91083, -79.05208	10.42	No	310		87
14. Garrison Rd. East		42.90378, -79.05497	7.50	Yes	370	215	54
15. Gorham Rd		42.89074, -79.05962	6.80	No	17		0
16. Nigh Rd		42.8938, -79.05718	5.11	No	25		0

In fall 2011, drain maintenance activities were undertaken by the municipality on a 1 km segment of the western reach, approximately 4 km upstream of the confluence of the west and south branches. Drain maintenance included vegetation removal from the channel, deepening of the channel, and construction of six remediation pools with terracing for the re-establishment of riparian vegetation [33]. During the Beaver Creek maintenance activities, modifications to the channel-thalweg were designed to increase sinuosity and add in-channel features, using anchored large woody debris, rocks to create scours, and undercuts for fish habitat [76]. These modifications differ from most drain maintenance activities that decrease sinuosity and remove in-channel features. In anticipation of these drain maintenance and remediation activities, fish habitat monitoring, and Grass Pickerel tracking protocols were planned and implemented in advance of the drain maintenance.

### Habitat and fish surveys

Aquatic habitat and the fish community of Beaver Creek were surveyed monthly by DFO from spring (April or May) to fall (November) from 2009 to 2013, with a few late surveys in 2015, at 10 core sites spanning the watershed [10]. Infrequent surveys were also undertaken at six intermediate sites in the watershed. During surveys, the fish community was sampled with 3–5 seine hauls using a 9.1 m bag seine with 3.2 mm mesh. The total count of each fish species captured was recorded during seine hauls, and all Grass Pickerel were measured for total length (mm), weight (g), and scanned with a PIT tag detector (Oregon RFID, Portland Oregon, USA) to check if the fish was tagged. For tagged fish, total length, and weight were recorded, and the tag ID was marked as recaptured. Habitat variables measured at each sampling site included water-chemistry variables (water temperature and conductivity) and stream morphology (stream width, pool depth, and percent vegetation channel cover). Water chemistry variables were measured with a YSI 6600 Surveyor 4a sonde, and all other measurements were made using standard measurement methods [10].

### Grass Pickerel tagging and tracking

Grass Pickerel greater than 180 mm TL captured during seining were implanted with a PIT tag (23 mm HDX, Oregon RFID; [10]. The tagging procedure involved anesthetizing Grass Pickerel in a clove oil bath to stage 3 anesthesia (partial loss of equilibrium,[39], placing fish in a moistened surgical sling, and making an approximately 5 mm incision just behind the pelvic girdle using a sanitized surgical blade. Sanitized PIT tags were inserted anterior to the body cavity, and the surgical incision was closed with a monofilament suture. Fish were periodically irrigated with creek water during the surgery to maintain ambient (approximately 15–22 °C) temperature and dissolved-oxygen concentration. After the surgery, Grass Pickerel were placed in a flow-through recovery tank in the stream, monitored for 1 h for proper swimming activity, then released back to the capture site.

Tagged Grass Pickerel were monitored throughout the watershed at eight antenna stations, with upstream catchment areas ranging in size from 9.35 to 37.3 km^2^. Antennas were distributed between 1.38 and 6.88 river km apart from one another, with four antennas on the west branch, two antennas on the south branch, and two on the mainstem (Fig. 1; Table 1). Antenna stations had six primary components: welding-cable antenna loops, T-bar support rods, tuner boxes, twin-axial cables, 2–12 V marine batteries, and multiplexers. Antenna stations were set up such that wire loops crossed the bottom of the creek perpendicular to the flow direction and across the entire channel area. Antennas were optimized for PIT-tag detection within a 0.5 m range of the antenna and tested by passing a dummy tag at multiple distances from the array. Under most stream conditions, the 0.5 m depth was sufficient to ensure any tagged fish moving upstream or downstream would be detected, although it is possible during extreme flood stages fish might have been able to pass a distance > 0.5 m from an antenna. Where possible, antenna wires were placed adjacent to culvert openings or other flow restrictions so there would be a lower likelihood of Grass Pickerel swimming around an antenna and avoiding detection. Antenna loops were anchored with T-bars and connected to tuner boxes (Standard Tuner Box, Oregon RFID) at the side of the creek. Twin-axial cables connected the tuner boxes to the multiplexer and power unit, which were located nearby in a tree stand to prevent water intrusion. Tuner boxes were calibrated biweekly in spring, summer, and fall months based on the availability of field personnel and tuned to a ~ 0.3 to 0.5 m optimal tag detection range with a test tag. While Grass Pickerel spawning is not well characterized, it has been suggested that Grass Pickerel utilize flooded terrestrial vegetation as spring spawning habitat [41, 72]. Given the study design to measure upstream and downstream in-channel movements, antennas were not optimised to measure potential lateral spawning movements to flooded vegetation, perpendicular to the primary channel direction. For each tagged Grass Pickerel that passed a detector, the tag ID number, time of detection, and the antenna station name were recorded on the multiplexers. Data were downloaded weekly from antenna stations during the tracking season (April or May to November from 2009 to 2013).

### Movement dataset assembly and analysis

Once movement data were collected from antenna stations and integrated into a single dataset, Grass Pickerel were determined to be stationary or mobile if their tags were detected at more than one station. Based on the organization of sampling sites and PIT antennas, Grass Pickerel had to move at least 500 m in-channel distance to be considered mobile (Fig. 1). For each mobile Grass Pickerel, a time-series detection history was generated for each fish, and all transitions between detections at two separate stations were treated as a movement. For these transition movements, movement distance is described as the stream distance between the two antenna stations, movement duration is defined as the time between the last detection at the first station and the first detection at the second station. For Grass Pickerel that made more than one movement, total movement distance was calculated as the sum of all movements made by that individual.

To calculate the adjusted stationary and mobile population proportions, a simple missed-tag correction was made based on non-adjacent site movements. In the movement dataset, several movements occurred between non-adjacent antenna stations, meaning that a Grass Pickerel passed an antenna station without detection. To make the best estimation of the number of real movements in the sample, the following adjustment was used:

$$Proportion \, Mobile= \frac{{N}_{mob} + {N}_{mob} * \frac{{M}_{n-adj}}{{M}_{adj}} }{{N}_{det}+ {N}_{det} * \frac{{M}_{n-adj}}{{M}_{adj}}} ,$$

where *N*_*mob*_ is the number of mobile individuals, *N*_*det*_ is the number of detected individuals, *M*_*n-adj*_ is the number of non-adjacent movements, and *M*_*adj*_ is the number of adjacent movements. In addition to the estimated total stationary and mobile population proportions, observed mobile, observed stationary, and untagged classifications were assigned to all Grass Pickerel in the five-year dataset and used as categories for subsequent analysis.

Kernel density plots were created for both the age distribution of Grass Pickerel captured (tagged and untagged) and the total distance traveled by mobile Grass Pickerel [26]. For mobile Grass Pickerel, the total distance traveled was calculated as the sum of individual movements made throughout the 2009 to 2013 study period. Grass Pickerel age was determined by the length-age relationship as established by Colm et al. [8] and kernel density estimation smoothing parameters were set to 0.66 to stabilize variation [6]. The shape of age distributions was compared between years by skewness, kurtosis, and one-sample Kolmogorov–Smirnov tests [18].

Fish condition for all tagged and untagged Grass Pickerel was calculated with Fulton’s *K*-value [29] and updated throughout the time-series dataset if a fish was recaptured. Then, condition values were grouped and compared by movement status (tagged mobile, tagged stationary, and untagged) and by year. The condition values were transformed in *z*-scores to minimize the unequal variances in the condition values through the years (Additional file 1: Table S1). Differences in fish condition z-scores by year and movement status were assessed using a two-way ANOVA correcting for the unbalanced design using a sum of squares type III model. The average of the total length of Grass Pickerel was also tested between movement status and between years using a two-way ANOVA with a sum of squares type III model to account for the unbalanced numbers of fish per year [73].

Grass Pickerel movements were mapped to show total counts of movements and movements per capita. In both cases, each movement in the dataset was assigned to the stream segment (here, a stream segment is defined as the channel distance between adjacent antenna stations) or segments it traversed. For movements made per capita, the average survey abundance of Grass Pickerel for the corresponding time period at the site on each end of a stream segment was averaged and then the movement count was divided by this average abundance. Mapping was generated using R *ggmap* package (R core team 2020). Total movement counts and per capita movements were compared spatially by branch (north, south, west) and by year using Kruskal–Wallis rank sum tests as data were non-normally distributed.

To perform a comparison between movement and habitat data, a subset of the data was used where habitat survey data overlapped with data for the continuous automated movement from the PIT tag sensors. Specifically, a 14-day window before and after each habitat sampling event (28 days in total) was used as the best approximation of how habitat conditions could affect the detected movements. Four different types of movements were considered: (1) movements to a site; (2) movements from a site; (3) the number of stationary tags at a site; and (4) the number of mobile tags at a site. There were 195 habitat survey events from 2009 to 2012 over 16 sites where water temperature, conductivity, channel vegetation coverage, and Grass Pickerel relative abundance were measured. From these 195 survey events, only 65 of them occur within the 14-day sample window where movements were detected (Additional file 1: Table S4).

The relationship between fish movement and habitat variables was assessed using two redundancy analysis models (RDA; [46], first focusing on immigration and emigration movements at a site, and second on the of number mobile and stationary tags at a site. Constrained ordination (RDA) was chosen over unconstrained ordination (PCA) to test the specific effects of a set of habitat variables on a set of movement variables, rather than test for variation within the whole sample. RDA analyses were performed in R (R vegan package), and variables were natural-log transformed to better meet assumptions of normality where appropriate (site immigration data, site emigration data, number of stationary tags, number of mobile tags, pool vegetation cover) consistent with Shen et al. [74] and Legendre and Legendre [46]. The performance of RDA models was evaluated with proportions of constrained variance and adjusted *R*^2^ values for the full models [62]. Lastly, site emigrations were regressed against site survey abundance to determine if sites with more Grass Pickerel correlated with a greater number of emigrations from the site using the same dataset. The significance of this regression was assessed with *Spearman ρ* rank correlation as there was unequal variance along the regression.

## Results

### Number and age distribution of Grass Pickerel

The total number and age distribution of Grass Pickerel captured during sample surveys varied between years (Additional file 1: Fig. S1; Table 1). The greatest number of Grass Pickerel captured was in 2009, with 2020 individuals captured during sampling. Local abundance was considerably lower in 2010 with 1137 Grass Pickerel captured during sampling, but this catch number was still higher than in subsequent years. During the 2011 and 2012 sampling surveys, 406 and 552 Grass Pickerel were captured, respectively. Presumed local abundance of Grass Pickerel dropped sharply in 2013 with only 72 Grass Pickerel captured. Grass Pickerel catch per unit effort (CPUE) varied considerably across the study period. The highest average catch per seine haul was in 2009 with an average of 17.6 Grass Pickerel caught per seine haul and the lowest in 2013 with an average of 1.70 Grass Pickerel captured per seine haul. During 2010, 2011, and 2012, an average of 7.25, 6.45, and 3.23 Grass Pickerel were captured per seine haul, respectively.

Mean age of Grass Pickerel increased from 2009 to 2011 and differed significantly between years (Type III ANOVA, *F* = 174.02, *df* = 1, *p* < 0.001). The mean age of Grass Pickerel was lowest in 2009 at 3.48 years and increased to 4.02 years and 4.64 years in 2010 and 2011, respectively. Younger Grass Pickerel were captured in 2012 and 2013 sampling surveys with mean age dropping to 4.06 and 3.48 years, respectively, and the distribution becoming more bimodal in 2013 (Additional file 1: Fig. S1; Table 2). The age-distribution shape varied from a normal distribution in all years (Additional file 1: Fig. S1; Table 2), with kurtosis values of 5.70 in 2009 and 7.39 in 2010 (Table 2). Of all the fish captured, 30 individuals were large (> 250 mm TL), which may have been 8-years of age if published Von Bertalanffy growth curves hold for all Grass Pickerel in the sample [8].

**Table 2:** Grass Pickerel age distribution in Beaver Creek (Ontario) from 2009–2013

Year	n	Mean length (mm)	Mean age (years)	SD	Skewness	Kurtosis	D-Value (KS test)	*p* (KS test)
2009	2020	169	3.48	1.13	1.26	5.70	0.93	< 0.001
2010	1137	181	4.04	1.21	1.12	7.39	0.93	< 0.001
2011	406	194	4.64	1.33	-0.27	-0.45	0.76	< 0.001
2012	552	182	4.05	1.82	0.61	0.44	0.81	< 0.001
2013	72	174	3.70	2.14	1.17	0.06	0.50	< 0.001

#### Tagged stationary and mobile Grass Pickerel

**Fig. 2:**
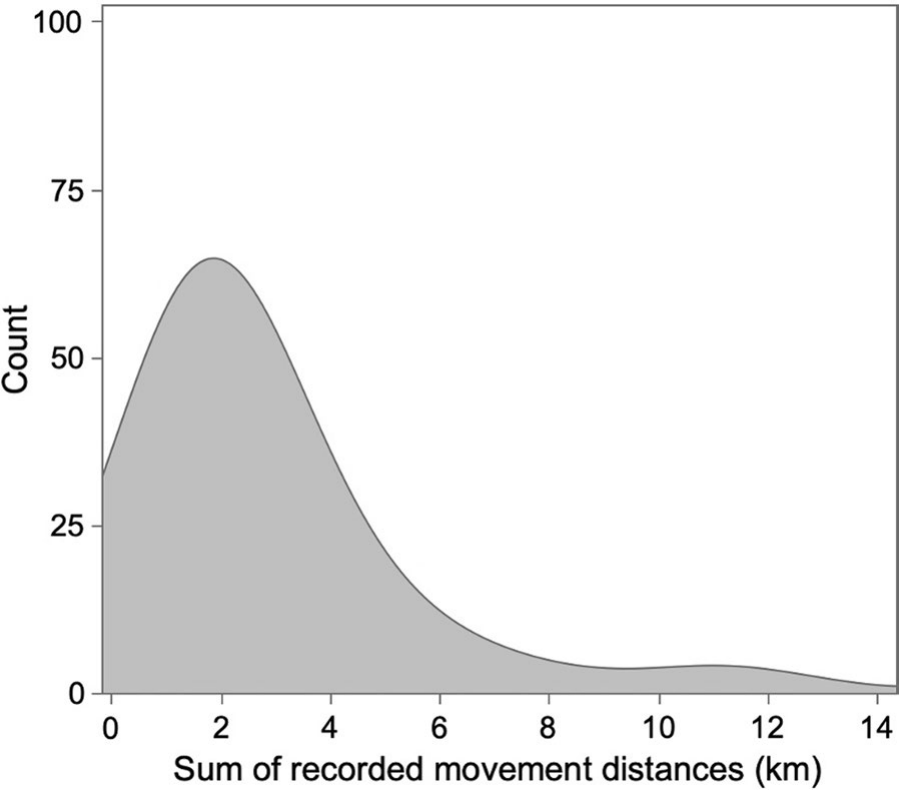
Kernel density plot of recorded Grass Pickerel movement distances for tracking history of each individual (*n* = 171) in Beaver Creek, Ontario, 2009–2013. For fish that made more than one movement, the sum of their movement distance was plotted. Movements ranged from 0.43 to 13.50 km with a median movement of 1.89 km

A total of 2007 Grass Pickerel were surgically implanted with PIT tags from 2009 to 2013 (Table 1). Of the tagged fish, 1074 tags were detected by the PIT antennas (Table 1). A total of 171 (8.5%) tagged Grass Pickerel were mobile or detected at more than one site over the sample period. Mobile Grass Pickerel made a total of 228 movements where a movement was defined as the least amount of time between detections at two separate sites. 47 individual Grass Pickerel made more than one movement during the study period. After the missed tag movement adjustment, approximately 15.9% of the sampled fish were mobile (Additional file 1: Fig. S3). Detected Grass Pickerel movements ranged from 0.43 km to 13.50 km with a median movement distance of 1.89 km (Fig. 2). Movement duration typically ranged from under a day for some short (< 1.12 km) movements to some movements that occurred over a year-long period, yet most movements (87.7%) occurred in less than 365 days. There were two instances of aggregate movements (14/09/2009 and 10/07/2010), with large numbers of Grass Pickerel moving from one site to another in a window of a few days or less (Additional file 1: Fig. S5).

#### Grass Pickerel condition and length

**Fig. 3:**
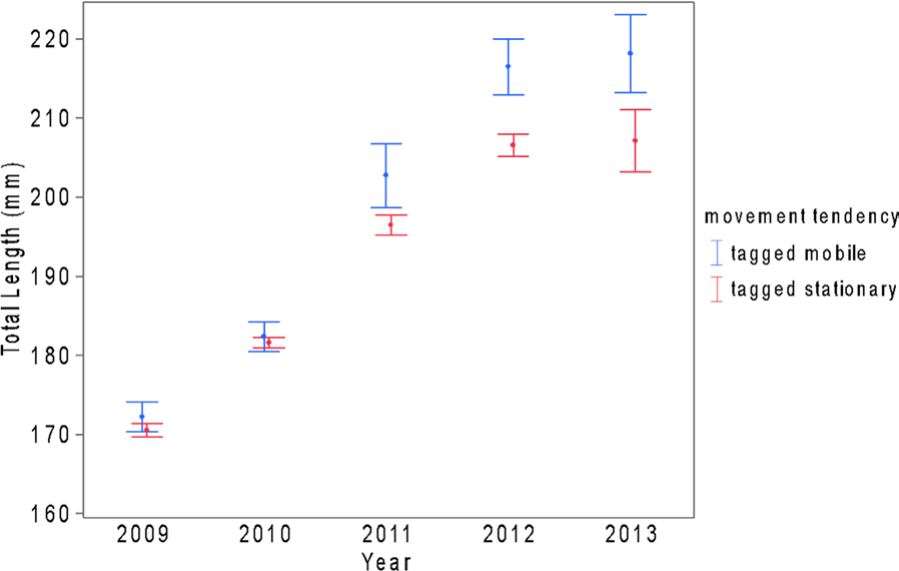
Total length (mm) of stationary and mobile population proportions for Grass Pickerel in Beaver Creek (Ontario) from 2009 to 2013. Each point denotes the annual mean length, and error bars are presented using one standard error. Difference in length is significant between years as well as between movement tendencies

Grass Pickerel condition did not significantly differ between tagged stationary and mobile fish (Additional file 1: Fig. S4; Table S3). However, condition varied significantly across years (Additional file 1: Fig. S4; Table S3; Type III two-way ANOVA, *F* = 40.88, *df* = 4 and 2410, *p* < 0.001) where condition values were significantly lower in 2012 than 2009–2011 (Tukey’s post hoc test: all *p* < 0.001). Grass Pickerel average length varied significantly between years (Fig. 3; type III two-way ANOVA, *F* = 204.2, *df* = 4 and 2410, *p* < 0.001) and was significantly higher for mobile tagged fish than stationary fish (Fig. 3; type III two-way ANOVA, *F* = 5.136, *df* = 1 and 2413, *p* = 0.024).

#### Spatio-temporal patterns of movement

**Fig. 4:**
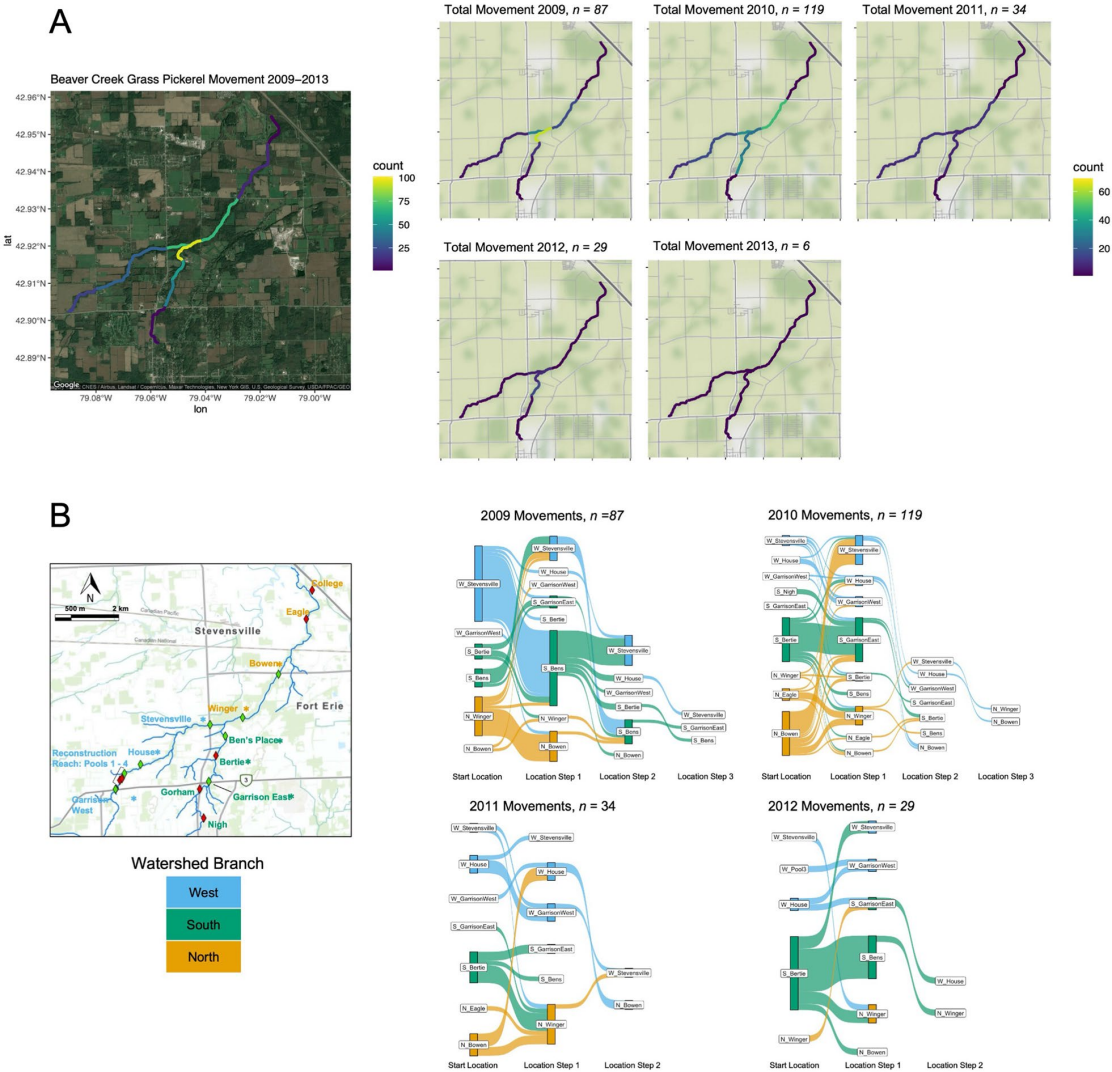
**A** Heatmap of Grass Pickerel movements in Beaver Creek (Ontario), summarized from 2009–2013 and by year. **B** Alluvial plot of movements made by Grass Pickerel in Beaver Creek, 2009–2012. Movements made in 2013 were not included as an alluvial plot as there were few (*n* = 6) movements made. Nodes represent sites, with advancing stages representing subsequent (and different) site detections. Node size is scaled to the number of movements started or ended from the site, and the color corresponds to the branch of the watershed

Grass Pickerel movements occurred in all branches of the watershed, with the greatest numbers of movements near the confluence of the western and southern branches of the creek (Fig. 4a; 95 movements recorded between the Winger Rd. and Ben’s Place antenna stations). The spatial organization of Grass Pickerel movements per capita differed slightly, with more even movement per capita recorded throughout the watershed compared to the total number of movements (Additional file 1: Fig. S6). Despite the differences in movement counts per branch, Kruskal–Wallis rank sum tests only revealed significant relationships between the number of movements and year (Fig. 4a; *n* = 60, Chi-square = 22.59, *p* < 0.001), but no relationship between the number of movements and watershed branch. Movements per capita revealed the same relationship, with significantly more movements per capita in 2010 and 2011 (Additional file 1: Fig. S6; *n* = 60, Chi-square = 16.10, *p* = 0.003), but no relationship between movement per capita and watershed branch. Moderate to low movement counts in all years of sampling (both before and after drain reconstruction) were recorded between Garrison West Road and Pools 1–4 (i.e., the reconstructed reach). There were no clear trends between the day of year and the date of movement initiation (Additional file 1: Fig. S7).

**Fig. 5:**
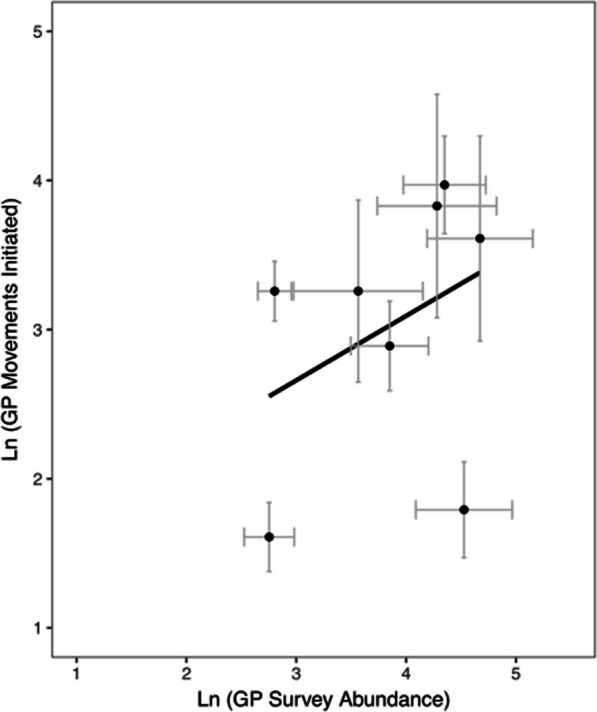
Number of movements initiated from a site plotted against Grass Pickerel survey abundance at that site in Beaver Creek (Ontario) from 2009 to 2013. Each point represents the mean values of movements initiated and survey relative abundances for a site (Additional file 1: Table S4; *n* = 65). Error bars denote one standard error for the transformed number of emigrations or survey abundance for each site over the sampling period. The significant, positive relationship between these variables indicates that Grass Pickerel are more likely to emigrate from sites with high survey abundances

There was a significant positive relationship between the number of movements initiated from a site (emigrations) and the Grass Pickerel abundance at that site (Fig. 5; *n* = 65, *Spearman ρ*: 0.498, *p* < 0.001), although there were unequal variance values for the number of movements initiated and survey abundance for sites used in the regression (*R*^2^ = 0.21).

#### Redundancy analysis of fish movement and habitat data

**Fig. 6:**
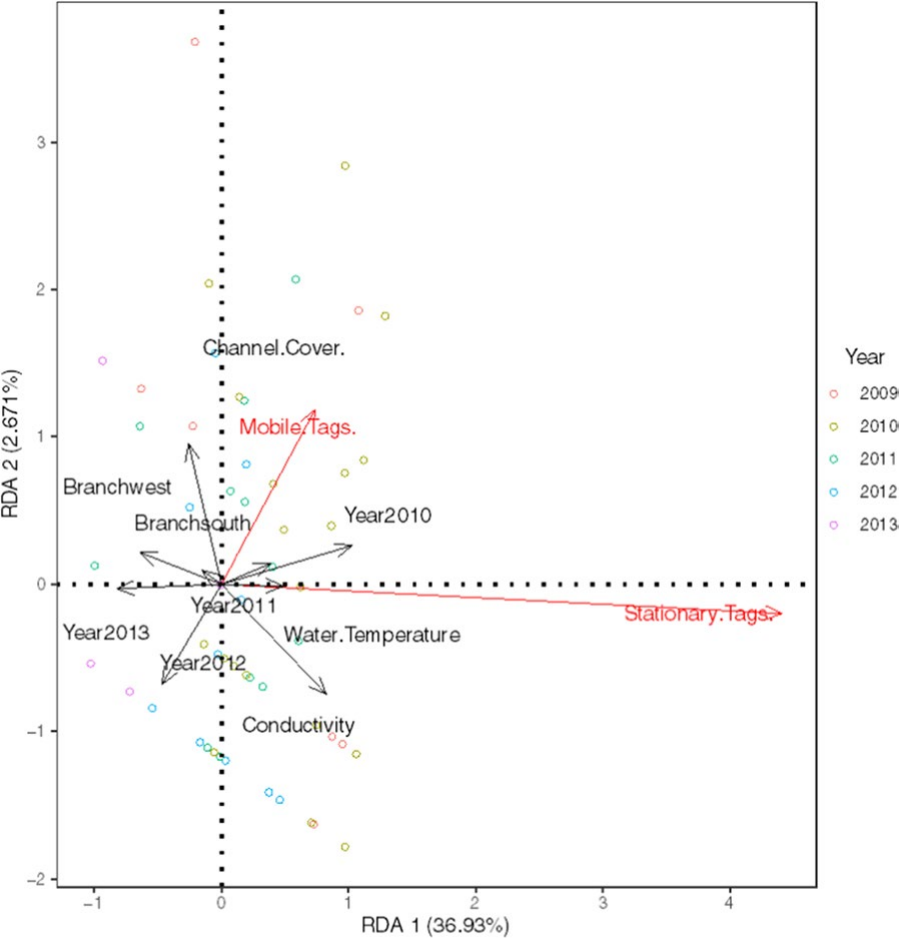
Redundancy analysis of Grass Pickerel movements (stationary and mobile tagged fishes) and habitat variables at surveyed sites in Beaver Creek, Ontario, 2009–2013. Automated recording of the number of mobile tags and stationary tags was summed for two weeks before and after a site observation where habitat variables (vegetation cover, conductivity, water temperature) were measured (*n* = 65). The number of stationary and mobile tagged data and channel vegetation cover data were natural-log transformed to meet assumptions of normality. Observations with either zero mobile or stationary tags, or outlier values were removed prior to RDA analysis. The two outlier points scoring high on RDA axis 2 were validated as site visits with high relative abundances of mobile tags

Through the sampling period (2009–2013), there was considerable variation in water temperature, conductivity, and channel vegetation measurements between years and sites. Generally, sites on the mainstem had higher vegetation coverage than those in the western or southern branches (Additional file 1: Table S2). There were generally higher average water temperatures recorded in 2010, 2011, and 2012 than in other years of sampling (Additional file 1: Table S2), and the Garrison East site consistently had higher conductivity values than other sites in the watershed. The redundancy analysis between the fish movement to and from sites (number of emigrations and number of immigrations) and environmental variables (temperature, vegetation coverage, conductivity, stream branch, and year) had poor explanatory power where 9.47% and 4.51% of the variation in the response variables was explained by the first two axes, respectively (Additional file 1: Table S5; Fig. S8). The RDA with the number of stationary and mobile tags as response variables and the same environmental variables had higher explanatory power with 36.90% and 2.67% of the variation in the response variables explained by the first two axes (Additional file 1: Table S5; Fig. 6; adjusted *R*^2^ = 0.297). Generally, the number of stationary tags, conductivity, water temperature, and 2010 samples had positive scores on the RDA1 axis. The number of mobile tags and channel vegetation cover tended to score positively on the RDA2 axis. Despite the improvement in constrained variation explained in the second RDA, the low *R*^2^ implies that much of the variation in mobile and stationary tag numbers is unexplained by the selected environmental variables.

## Discussion

Grass Pickerel movement in an agricultural drain varied, with small proportions of the sampled fish undertaking movements greater than 500 m. Movements were more likely to initiate from sites with high local abundances. Stationary and mobile proportions of sampled fish exhibited slight differences in size, with mobile fish having a greater total length on average, perhaps in support of a pace of life syndrome advantage (hereafter, PoLS) or an increased survival rate [45, 52, 87]. Variance in the stationary and mobile population proportions between sites was better explained by habitat variables than the number of immigrations or emigrations between sites. The distribution of Grass Pickerel movement distances showed a long-tail distribution, implying high intraspecific movement variation among the studied Grass Pickerel, which may have implications for gene flow among both historical and contemporary Grass Pickerel populations [50]. Low frequency of movements over 5 km and low frequency of movements downstream from Beaver Creek to Black Creek are indicative of difficult movement conditions between the Beaver Creek agricultural drain and other neighboring habitats.

### Grass Pickerel movement

In the Beaver Creek system, Grass Pickerel were generally stationary, with approximately 16% of the sampled fish making movements greater than 500 m. When Grass Pickerel moved, movements were more likely to originate from sites with high local abundances (Fig. 5), and movement was less prevalent in years with lower Grass Pickerel local abundances. If high local Grass Pickerel abundance induced movement, then Grass Pickerel density would likely have had a strong positive relationship with movement probability. Despite a significant, positive relationship between emigrations and site relative abundance (Fig. 5), the regression between these variables had a relatively low *R*^*2*^ value with considerable unequal variance, which may imply either a nonlinear or variable relationship between site emigrations and local Grass Pickerel abundance. More frequent movements from sites with high abundances were generally consistent with density-dependent movement tendencies. It is possible that detected Grass Pickerel movements from sites with high local abundances were in direct response to resource depletion or competition, as fish foraging decisions are often influenced by competition, and the density of Grass Pickerel in Beaver Creek was very high in the early years of the study [10, 12]. Further, the long right-tail movement distance distribution matched that of many other species with a long right-tail dispersal kernel [40]. While the bulk of movements made by species with long right-tail dispersal kernels is very short, these long-distance dispersal events (or highly mobile individuals) are of ecological importance to facilitate genetic connectivity, for (re)colonization of vacant habitats, and recovery from local extirpations [28, 68].

Mobile Grass Pickerel were longer than stationary fish by a mean of 4 mm across all years, and by about 10 mm during 2012 and 2013 (Additional file 1: Table S1). This equates to roughly 5% greater total length for mobile Grass Pickerel. According to the PoLS hypothesis, mobile fishes tend to achieve a slightly greater size at age than stationary counterparts (approximately 5–10% greater length in Northern Pike) through a faster pace of life and an associated elevated metabolism [45]. The greater length for mobile fishes is generally believed to be an effect of metabolic optimization, mobile fishes can access more metabolically optimal habitats and achieve greater body size for a given age [4]. However, in the Beaver Creek system, which experienced a sharp reduction in abundance over the study period, mobile Grass Pickerel may have survived longer and thus achieved a greater length than stationary Grass Pickerel that perished earlier in their lifespan. If this was the case, mobile Grass Pickerel would exhibit enhanced survival compared to stationary Grass Pickerel, but there would not necessarily be a growth rate or PoLS benefit for mobile fish. Decoupling these two effects would require a more intensive investigation of growth rates (e.g., length at age) or survival rates of stationary and mobile Grass Pickerel populations. While a length-at-age Von Bertalanffy curve has been published for Beaver Creek Grass Pickerel [8], it is calculated from Grass Pickerel throughout Beaver Creek and does not differentiate by site or movement status (though there is some variation in growth rate by year). Intermittent lethal sampling to validate the Colm et al. [8] growth curve may be possible, or it may also be feasible to perform a catch-curve analysis to infer survival differences between stationary and mobile population proportions [77]. Regardless of PoLS or survival effects, our results demonstrate that mobile Grass Pickerel likely achieved a fitness benefit through movement, as shown by greater body size.

Over the study period, a drought in the summer 2012 was believed to affect the relative abundance and condition of Grass Pickerel, as well as potential movement connectivity among sites [10]. However, the results of the RDA showed little relationship between movement and habitat variables (Fig. 6). There may be several explanations for this mismatch between environmental conditions and patterns in the movement data including low spatio-temporal resolution of habitat monitoring data (biweekly or less frequently, taken at discrete sites ~ 500 m apart), or reduced movement signals as a result of sharp declines in the Grass Pickerel population.

To better characterize movement responses to habitat change, wetted habitat profiles of the stream that show where and when fragmentation occurred in the watershed with a high spatial and temporal resolution would help quantify movement responses pre- and post-fragmentation. With the antennae and habitat sampling sites used in this study, it is likely that stream fragmentation occurred in reaches between antenna sites during the summer of 2012 (Julia Colm, Fisheries and Oceans Canada, personal observation). Monitoring designed to precisely characterize movements around fragmentation points during summer or low-flow conditions may provide greater insight into how Grass Pickerel move either in anticipation of, or in response to, drought conditions. High temporal resolution, such as daily monitoring of habitat conditions, is likely important for characterizing these drought-response movements as some stream fishes have shown high movement activity prior to fragmentation [23]. These movement responses to drought may be ecologically relevant for Grass Pickerel as they likely have an evolutionary history of utilizing habitat that is subject to frequent fragmentation [16], and many contemporary riverscapes experience these conditions more frequently as a result of climate change [82]. Likewise, more locally specific movement data for the stream segments that become isolated during drought conditions may also show a clearer signal between drought and small-scale movements. The scale of movements possible to detect in this study (> 500 m) may be larger than those relevant to locating drought refugia, which often operate as small, isolated habitat patches along stream reach during severe drought conditions [48].

During the two mass-movement events on 14/09/2009 (*n* = 23) and 10/07/2010 (*n* = 13), many Grass Pickerel initiated movement from one site and ended movement at a different site within a window of a few days (Additional file 1: Fig. S5). There were several possible explanations for these aggregate movements, including stochastic disturbances that encouraged movement such as a spill, social cues from movements made by other fish, or environmental thresholds in habitat quality (e.g., water temperature or dissolved oxygen content) being exceeded [38, 47, 86]. Given the limited prevalence of these aggregate movements in this study, it seems that aggregate movements were an uncommon movement type (e.g., disturbance response), rather than the norm for Grass Pickerel.

The trend of sharply decreasing local abundance may have made movement responses to drought or habitat alteration harder to detect. The widespread decrease in local Grass Pickerel abundance from 2009 to 2013 might have the effect of: (1) decreasing the total number of detectable movements made by Grass Pickerel (Fig. 4a); and (2) decreasing the need for movement away from habitats with high densities of Grass Pickerel, as local abundances were much lower throughout the system. Specifically, the movement signals from drought that might be anticipated (i.e., more movement as a stream de-waters, longer movements over unsuitable habitats) may be outweighed by the overwhelming regime-shift, or change in movement ecology, from the strong decrease in Grass Pickerel abundance.

### Comparisons between Grass Pickerel movement and other species

There are similarities between the movement tendencies documented and the movement of other freshwater predatory fishes. Extensive research on the movement of Northern Pike (*Esox lucius*) has shown diverse movement strategies across populations in Europe and North America, with stationary, diffusive, and migratory movement behaviours all demonstrated in riverine populations [44, 60, 71]. While Northern Pike habitat is typically deeper and, as a result, more perennially connected than Grass Pickerel habitat, one of the strongest parallels between the two species’ movement strategies is high intraspecific variation. Sandlund et al. [71] described a population of Northern Pike in a river-reservoir system where the bulk of Northern Pike moved under 2 km during their lifespan, but a few individuals made regular directional migrations up to 14 km [71]. The high intraspecific variation in movement tendencies found by Sandlund et al. [71] is similar to that observed in the Beaver Creek Grass Pickerel population and may indicate the evolutionary importance of diverse movement strategies within populations of predatory riverine fishes, as discussed in the fish movement ecology literature [11, 34, 67]. For individuals that successfully make long-distance movements (Grass Pickerel or other species), individual behaviour or ‘animal personality’ may well interact with opportune spatio-temporal habitat conditions to facilitate long-distance movements [34].

Intrinsic (or individual) differences between mobile and stationary fishes are demonstrated among many species and disparate systems (e.g., marine, lake, and riverine environments) and likely result from different foraging, survival, and homeostatic abilities associated with movement [12]. McKee et al. [52] demonstrated that mobile Walleye (*Sander vitreus*) achieved a greater asymptotic growth limit, and female Walleye thereby produced a greater number of eggs, by completing movements out of a protected bay habitat and into a deeper pelagic habitat. While there are considerable differences between the shallow stream habitat of Grass Pickerel in Beaver Creek and the open-water habitats utilized by Walleye in McKee et al. [52], the shared apparent fitness benefit (of enhanced length) supports the hypothesis that movement influences different fitness outcomes across predatory fishes. As tags and tracking systems improve to allow the tagging of smaller-bodied species [37], it may be possible to determine if similar movement-fitness relationships are shared by non-predatory fishes.

Comparing Grass Pickerel movement to movement studies on other threatened fishes reveals the common challenges in characterizing habitat variables important for movement. For focal populations in river systems with flow data at high temporal resolution (daily or hourly), specific flow events, such as short, elevated discharges, may encourage movement, but this likely depends on the biology of the species [81]. Experimental manipulations of flow volume have shown that some cold-water stream fishes (*Catostomus cf. catostomus* and *Oncorhynchus kisutch*) move towards specific, more oxygenated, refugia following decreases in flow volume [88]. However, the context of flow regime (and whether flow is altered by any human-made infrastructures) likely influences both the availability of ideal habitat and movement costs associated with accessing that habitat, as has been demonstrated by studies on movement in endangered Western Silvery Minnow (*Hybognathus argyritis*) and Rocky Mountain Sculpin (*Cottus* sp*.*) [59, 70]. Somewhat similar to our findings on Grass Pickerel, in a movement study on endangered Redside Dace (*Clinostomus elongatus*), some relationships were found between movement and environmental factors including stream depth, width, and volume, yet considerable amounts of variation in movement data were unexplained by environmental factors [21].

### Agricultural drain movement ecology

The results of this study indicate that Grass Pickerel is generally unlikely to move from suitable habitats unless high local abundances are reached. Prior research on Grass Pickerel has demonstrated the species to be a wetland-habitat specialist and more likely to occur at sites with specific features including high aquatic vegetation cover, high conductivity (evidence of a clay surficial geology), low bank slope, and large woody debris [9, 16]. As many sites in the Beaver Creek watershed contain these attributes, it may reduce the need for movement to locate suitable habitat areas (outside of specific disturbance events, like the drought of 2012). Moreover, broad emigration from the Beaver Creek system may be unlikely. Few Grass Pickerel tagged upstream were recaptured downstream at the Eagle and College sites, and there was limited movement downstream on the north branch of the watershed (at the Winger, Bowen, Eagle, and College sites) in general, implying that the population may be effectively confined to the upper reaches of the watershed (Fig. 4b). Likewise, immigration also appeared to be rare, with few Grass Pickerel tagged at College or Eagle moving upstream, indicating that recruitment to Beaver Creek from downstream sources (Black Creek) may be limited (Fig. 4b). An antenna station at the College site and tagging efforts in Black Creek near the confluence with Beaver Creek could have helped resolve immigration and emigration patterns. Likewise, as is consistent with any PIT tagging study, post-handling stress may affect the behaviour and survival of tagged fish [39]. The low immigration and emigration findings are consistent with a relatively stationary population that may disperse sporadically from sites with high local abundances.

For the few Grass Pickerel that successfully made long-distance movements in the Beaver Creek watershed, most movements (> 10 km) consisted of movement to a site in another branch of the watershed (i.e., west branch to north or south branch) and then a return movement to either the original site or a nearby site. The return-movement behaviour differed from the majority of the movements made by Grass Pickerel over the study period that were a single-step or involved movement to a new site with no detected return (Fig. 4b). The difference between single-step and return movements may have ecological significance, as single-step movements are routinely documented in riverine fish populations inhabiting varied riverscapes [15]. Single-step movements are often associated with dispersal events, nomadism or, in some cases when there are changes in habitat conditions, or a change in home range [1]. Return movements may serve different ecological functions, perhaps serving as habitat exploration, a larger home range, or an intermediate form of migration between optimal foraging, spawning, or rearing habitats [5]. Ultimately, more specific information on survival, growth, and fitness is needed to disentangle the functions of these different movement behaviours.

### Conservation implications

Grass Pickerel exhibits high intraspecific movement variation, which may serve multiple ecological functions relevant to its conservation. Among these ecological functions are the location of refugia during adverse conditions, colonization of new or reconnected habitat, effective feeding and predation avoidance, and maintaining metapopulation dynamics and gene flow among neighboring populations. Recent work on Grass Pickerel landscape genetics has shown limited to no gene flow over relatively short stream distances [50]. We have shown that a few Grass Pickerel may move relatively long distances (although none > 14 km), yet most tagged Grass Pickerel were stationary and fragmented riverscapes may prevent these long-distance movements from resulting in gene flow—consistent with the results of Lujan et al. [50]. If limited movement and, hence, gene flow, is similar in other Grass Pickerel populations throughout southern Ontario, there are a few key considerations for the conservation of the species.

First, Grass Pickerel populations may be on unique evolutionary trajectories influenced by their local environments. Our results support the broader understanding that Grass Pickerel populations separated by long distances (i.e., > 14 km) may represent discrete populations across their Canadian range [13, 50], but further, if Grass Pickerel is found to have different movement tendencies in other Canadian populations, it could be a sign of local adaptation and evolutionary significance. This could influence how disjunct Grass Pickerel populations are managed throughout their Canadian range, and what protections are applied to isolated populations. Hence, it is important to determine how applicable the movement results from this study are to other Grass Pickerel populations.

A second outcome of limited movement among Grass Pickerel populations may be risks of genetic isolation following disturbance. For populations that experience limited abundance or sharp decreases in local abundance (like the one documented in Beaver Creek over this study period), there may be adverse genetic effects from bottlenecking or reduced diversity if recolonization or genetic connectivity are not facilitated to neighboring populations [85]. Likewise, it is critical that future research investigates the causes of dramatic changes in local abundance, as preventing and managing these fluctuations may help preserve evolutionary processes for Grass Pickerel. The population threats from reduced genetic or allelic diversity are often increased as a result of greater disturbance from habitat degradation and climate change [49] and shared by many other threatened wetland species across southern Ontario [61, 66].

Future monitoring of Grass Pickerel movement could investigate movement tendencies in other Grass Pickerel populations across southern Ontario and revisit the Beaver Creek system to evaluate the viability of the population and the success of drain maintenance mitigations. Agricultural drain maintenance is relatively common across southern Ontario as it is legislated by the *Drainage Act*; prior work has shown fish communities to be somewhat resilient to drain maintenance activities, however, drain maintenance is likely more of a threat for imperiled fishes [33]. As stochastic disturbances, such as drought, may occur during, or immediately subsequent to drain maintenance activities, it is important to design drain maintenance to prioritize the stability and connectivity of key habitat throughout the maintenance process. While any direct effects on Grass Pickerel movement from drain maintenance were likely outweighed by the effects of drought and reduced population size during this study, movement did occur in the reconstructed reach (West Branch between Garrison West and Pool 1) both before and after maintenance activities, indicating that permanent fragmentation did not occur. More localized investigation of movement (for instance, 10’s of meters rather than 0.5 km) could further inform how Grass Pickerel, and potentially other wetland species, move around artificially altered channels and point disturbances. Using quantitative frameworks for assessing habitat quality, availability, and connectivity, such as those discussed in Montgomery et al. [55], will be critical to prevent deleterious effects from drain maintenance to Grass Pickerel and other imperiled fishes.

While we have shown that Grass Pickerel can move long distances under the right conditions, it is concerning that the genetic results of Lujan et al. [50] showed that these conditions are largely not met in current southern Ontario riverscapes. Historically, Grass Pickerel likely dispersed across the region from a single Pleistocene refugium and utilized abundant low-slope watershed or wetland habitats [51]. The drastic loss of wetland habitat due to widespread land use change in southern Ontario [61] has likely permanently altered this historical phenomenon. Given the high prevalence of agricultural drains in southern Ontario and other agricultural regions globally [36], understanding the mismatch of evolved movement ability and contemporary habitat conditions experienced by wetland specialist species may be key to preserving aquatic biodiversity in working agricultural landscapes.

## Conclusion

We demonstrate that Grass Pickerel in an agricultural drain system, Beaver Creek, are generally stationary, although there is considerable variation in movement tendencies, with some Grass Pickerel able to move relatively long distances. Mobile Grass Pickerel likely gain a slight fitness advantage, shown through a greater total length than stationary counterparts, and movements were more likely to originate from sites with high relative abundances. No clear relationship between movement variables and water temperature, channel vegetation, or stream conductivity was identified during the study, although there may be relationships between movement and unmeasured environmental metrics of drought. Our movement results also provide further support for considering Grass Pickerel populations as disjunct, as movement is largely limited between populations even at short stream distances.

The findings of our study could help manage the persistence of the imperiled fish species in agriculture drainages. As demonstrated, fish movement behaviour may not guarantee persistence during the most severe ecological and environmental conditions experienced in agricultural drainages. Continuing to develop knowledge on the range of habitat conditions experienced in agricultural drains and how imperiled species may respond to them will help conservation planning. Yet, further work is needed to mitigate riverscape fragmentation by undertaking habitat improvement to facilitate movement corridors between populations wherever possible.

### Supplementary Information

**Additional file 1**. Supplemental tables and figures.

## Data Availability

Data and analysis code will be made available upon request.
