# Supplementary material for: Movement of an imperiled esocid fish in an agricultural drain
Source: Mov Ecol. 2023 Dec 13;11:77. doi: 10.1186/s40462-023-00420-2 (PMC10720151; doi:10.1186/s40462-023-00420-2)
Supplement: Supplementary file 1 — Additional file 1. Supplemental tables and figures. [file 40462_2023_420_MOESM1_ESM.docx]

Supplement

**Table S1:** Condition and length mean values and variances for tagged mobile, tagged stationary, and untagged population proportions. Data collected from DFO Beaver Creek Grass Pickerel surveys, 2009 - 2013.

| *Year* | *Population Proportion* | *n* | *Mean Total Length (mm)* | *Variance (S^2^) Total Length* | *Mean Condition (Fulton’s K)* | *Variance (S^2^) Condition* |
| --- | --- | --- | --- | --- | --- | --- |
| *2009* | Untagged | 1661 | 159 | 1093 | 0.598 | 0.0194 |
|  | Tagged Stationary | 718 | 170 | 458.0 | 0.705 | 0.0282 |
|  | Tagged Mobile | 87 | 172 | 317.7 | 0.673 | 0.0363 |
| *2010* | Untagged | 154 | 109 | 1937 | 0.638 | 0.0399 |
|  | Tagged Stationary | 950 | 182 | 439.3 | 0.678 | 0.0076 |
|  | Tagged Mobile | 119 | 182 | 414.9 | 0.681 | 0.0047 |
| *2011* | Untagged | 305 | 121 | 4009 | 0.518 | 0.0625 |
|  | Tagged Stationary | 223 | 197 | 331.2 | 0.663 | 0.0084 |
|  | Tagged Mobile | 34 | 203 | 533.3 | 0.671 | 0.0039 |
| *2012* | Untagged | 391 | 137 | 1304.6 | 0.583 | 0.0133 |
|  | Tagged Stationary | 218 | 207 | 430.52 | 0.594 | 0.0099 |
|  | Tagged Mobile | 31 | 217 | 386.12 | 0.567 | 0.0053 |
| *2013* | Untagged | 155 | 78 | 1216.6 | 0.639 | 0.0198 |
|  | Tagged Stationary | 28 | 207 | 430.56 | 0.623 | 0.0144 |
|  | Tagged Mobile | 6 | 218 | 147.77 | 0.646 | 0.0004 |

**Table S2:** Summary of habitat conditions in Beaver Creek by year. All values are annual mean of full sample set (*n* = 195) and color scaled to the range of values experienced across Beaver Creek sampling 2009-2013.

**Table S3:** Type III two-way ANOVA results for Grass Pickerel condition *z*-score and length grouped by year or movement tendency.

| **Response Variable** | **Grouping** | **DF** | **Sum of Squares** | **F value** | **p** |
| --- | --- | --- | --- | --- | --- |
| **Condition Z-Score** | Year | 4 | 2.337 | 40.88 | < 0.001 |
|  | Movement Tendency | 1 | 0.026 | 0.161 | 0.689 |
| **Total Length (mm)** | Year | 4 | 350475 | 204.2 | < 0.001 |
|  | Movement Tendency | 1 | 2203 | 5.136 | 0.024 |

**Table S4:** Summary of movement-habitat sites (habitat surveys with no movements removed) in Beaver Creek, Ontario, 2009-2013.

| **Site** | **Years Represented** | **n surveys with coupled habitat -movement data** |
| --- | --- | --- |
| **Bowen Rd.** | 2009 - 2013 | 12 |
| **Winger Rd.** | 2009 - 2013 | 5 |
| **Stevensville Rd.** | 2009 - 2013 | 6 |
| **House Rd.** | 2010 – 2013 | 8 |
| **Garrison Rd. West** | 2009 - 2013 | 5 |
| **Ben’s Place** | 2011 - 2013 | 4 |
| **Bertie Rd.** | 2009 - 2013 | 13 |
| **Garrison Rd. East** | 2009 - 2012 | 7 |

**Table S5:** Movement-habitat RDA results for Grass Pickerel in Beaver Creek, Ontario, 2009-2012.

| ***RDA Candidate Model*** | ***Predictor Variables*** | | | ***Response Variables*** | | ***RDA1 Score*** | ***RDA2 Score*** | ***Constrained Variance*** | | | ***Adjusted R^2^*** | |
| --- | --- | --- | --- | --- | --- | --- | --- | --- | --- | --- | --- | --- |
| ***1: Emigration and immigration*** | • Conductivity | | | Movements Initiated | | -0.892 | 0.142 | 0.140 | | | -0.001 | |
|  | • Water Temperature | | | Movement Destinations | | -0.206 | -0.616 |  | | |  | |
|  | • Ln [Channel Cover] | | |  | |  |  |  | | |  | |
|  | • Stream Branch | | |  | |  |  |  | | |  | |
|  | • Year | | |  | |  |  |  | | |  | |
| ***2: Stationary and mobile tags*** | • Conductivity | | | Stationary Tags | | 2.204 | -0.991 | 0.396 | | | 0.297 | |
|  | • Water Temperature | | | Mobile Tags | | 0.368 | 0.593 |  | | |  | |
|  | • Ln [Channel Cover] | | | |  |  |  | |  |  | |  |
|  | • Stream Branch |  |  | |  |  |  |  |  |  |  |  |
|  | • Year |  |  | |  |  |  | |  |  | |  |


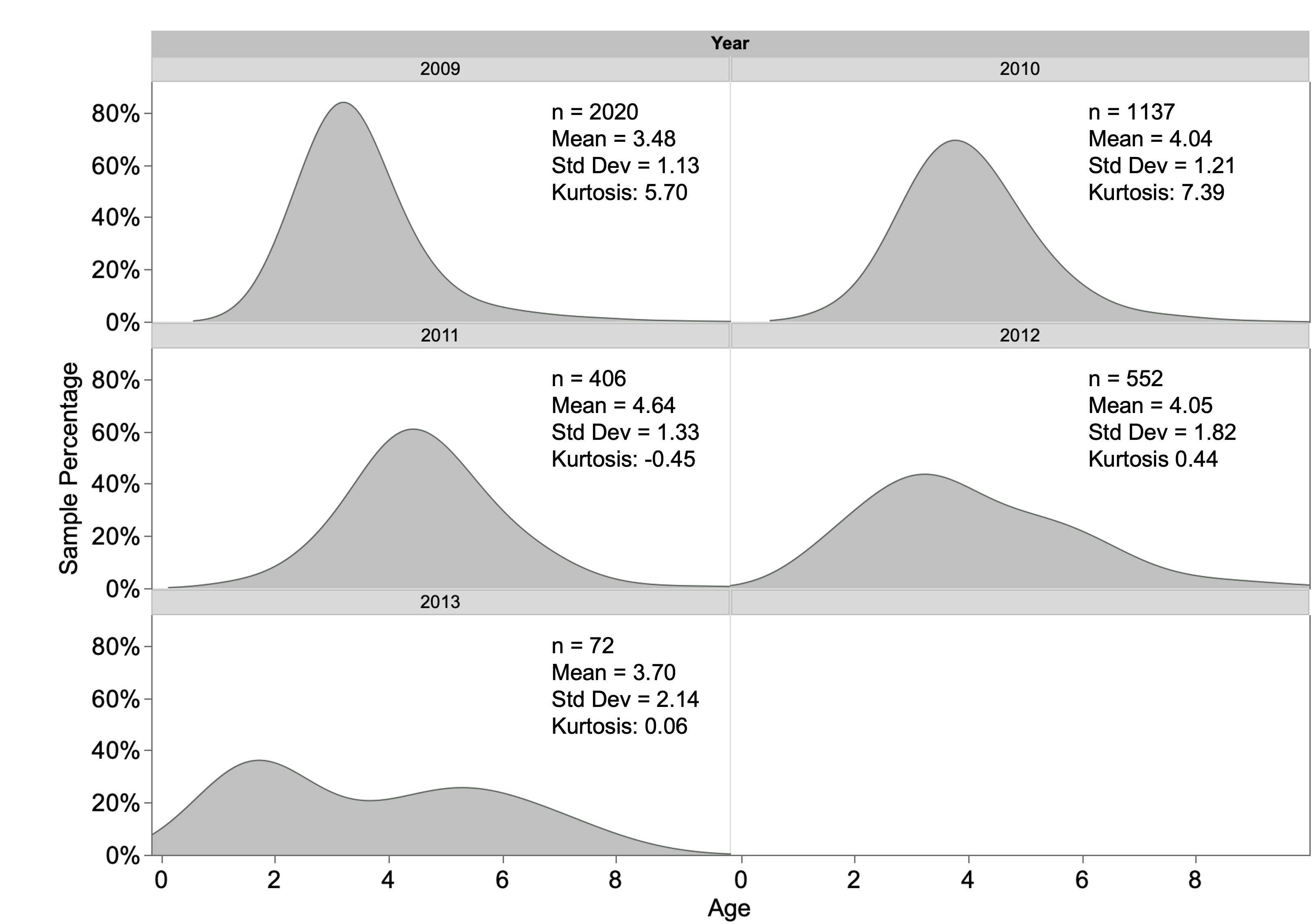


**Figure S1:** Age distribution of surveyed Grass Pickerel populations in Beaver Creek, 2009-2013. All ages were assigned using the Von Bertalanffy length-age curve for Grass Pickerel in Beaver Creek based on Colm et al. (2020).


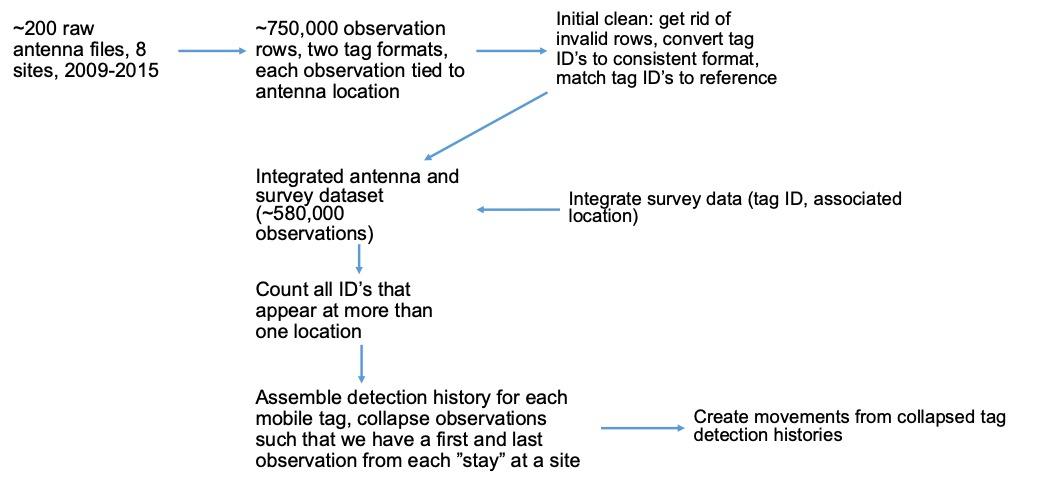


**Figure S2:** Flowchart describing data merging and cleaning to generate movement dataset from DFO Beaver Creek Grass Pickerel monitoring, 2009-2013.


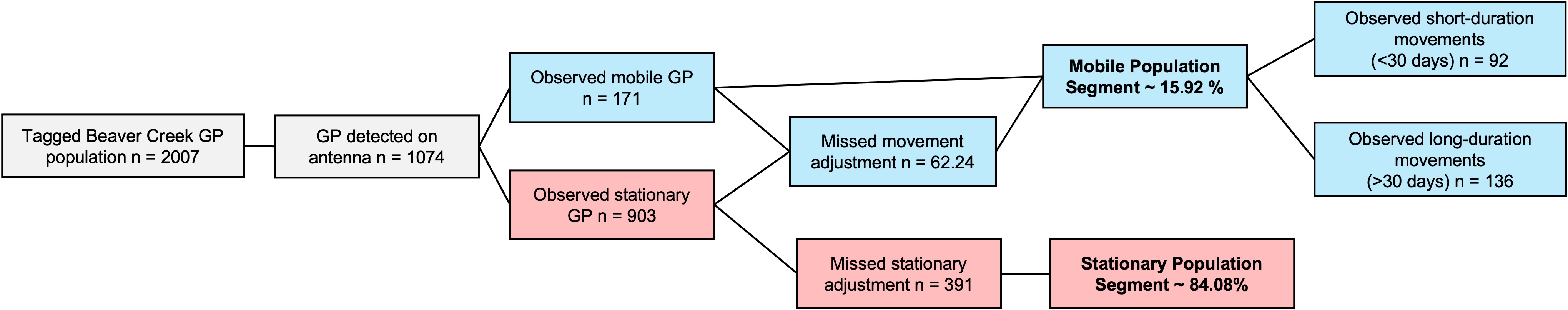


**Figure S3:** Flowchart describing assignment of stationary and mobile (> 500m movement) population proportions for Grass Pickerel in Beaver Creek, Ontario, 2009-2013.


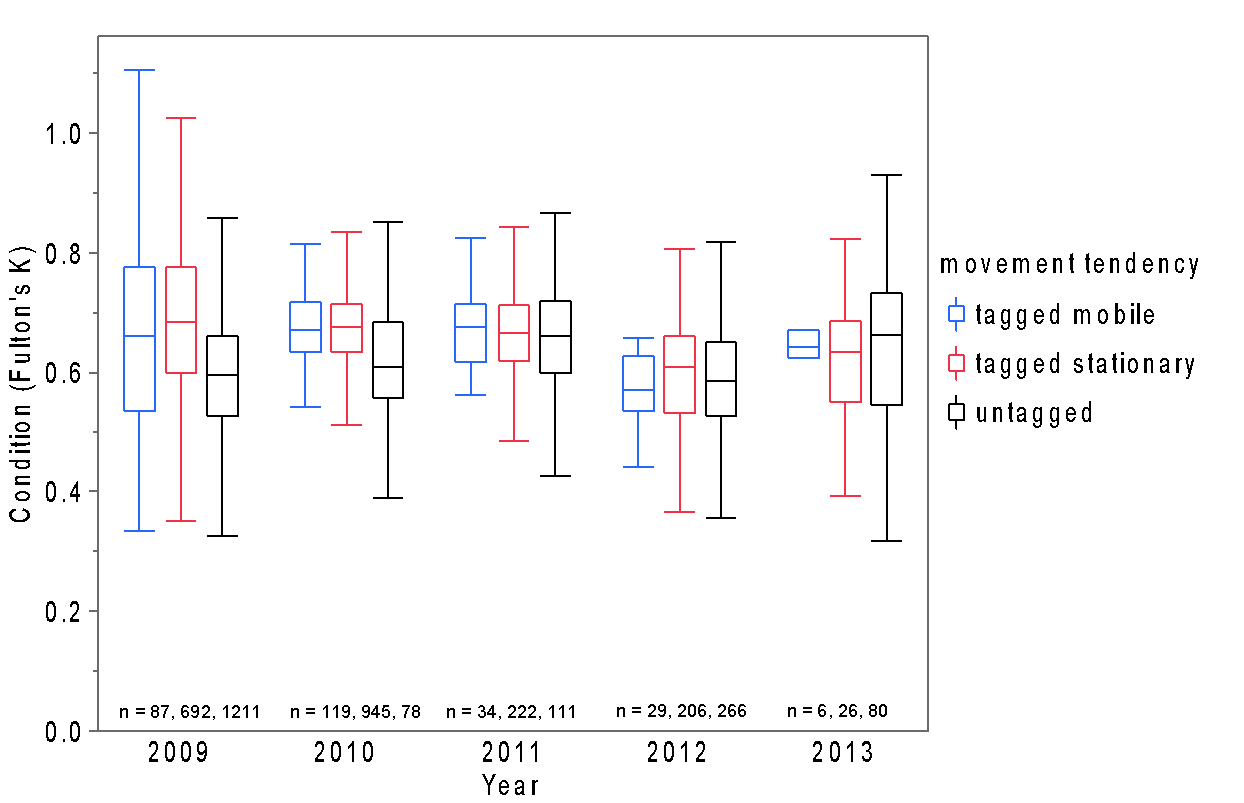


**Figure S4** Condition (Fulton’s *K*) of tagged stationary, tagged mobile, and untagged population proportions for Grass Pickerel in Beaver Creek, Ontario, 2009-2013. The center line of each boxplot denotes the median.

**
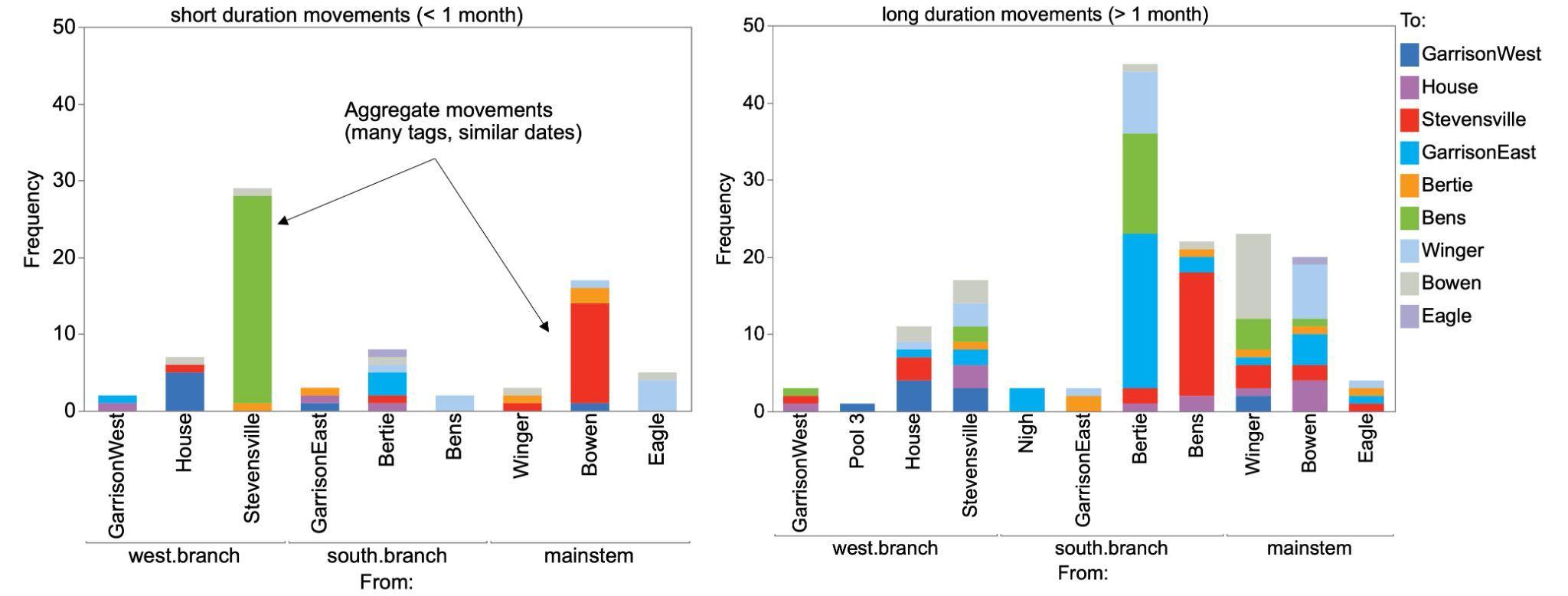
**

**Figure S5:** Movement origin and destination sites from movements made by tagged Grass Pickerel in Beaver Creek, 2009 to 2013. Origin sites are on the x-axis and destinations are denoted by color on the stacked histogram (*n* = 171).


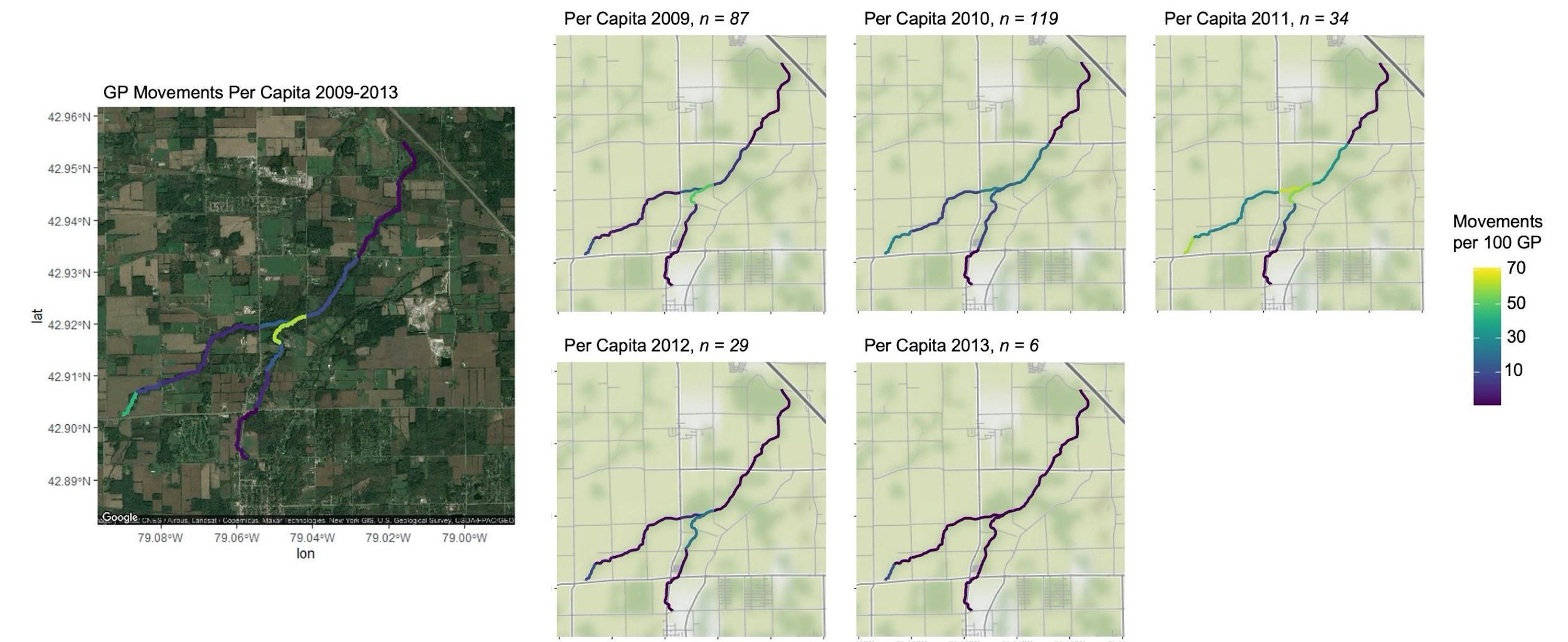


**Figure S6:** Heatmap of Grass Pickerel movements per capita site survey in Beaver Creek (Ontario), summarized from 2009-2013 and by year.

**
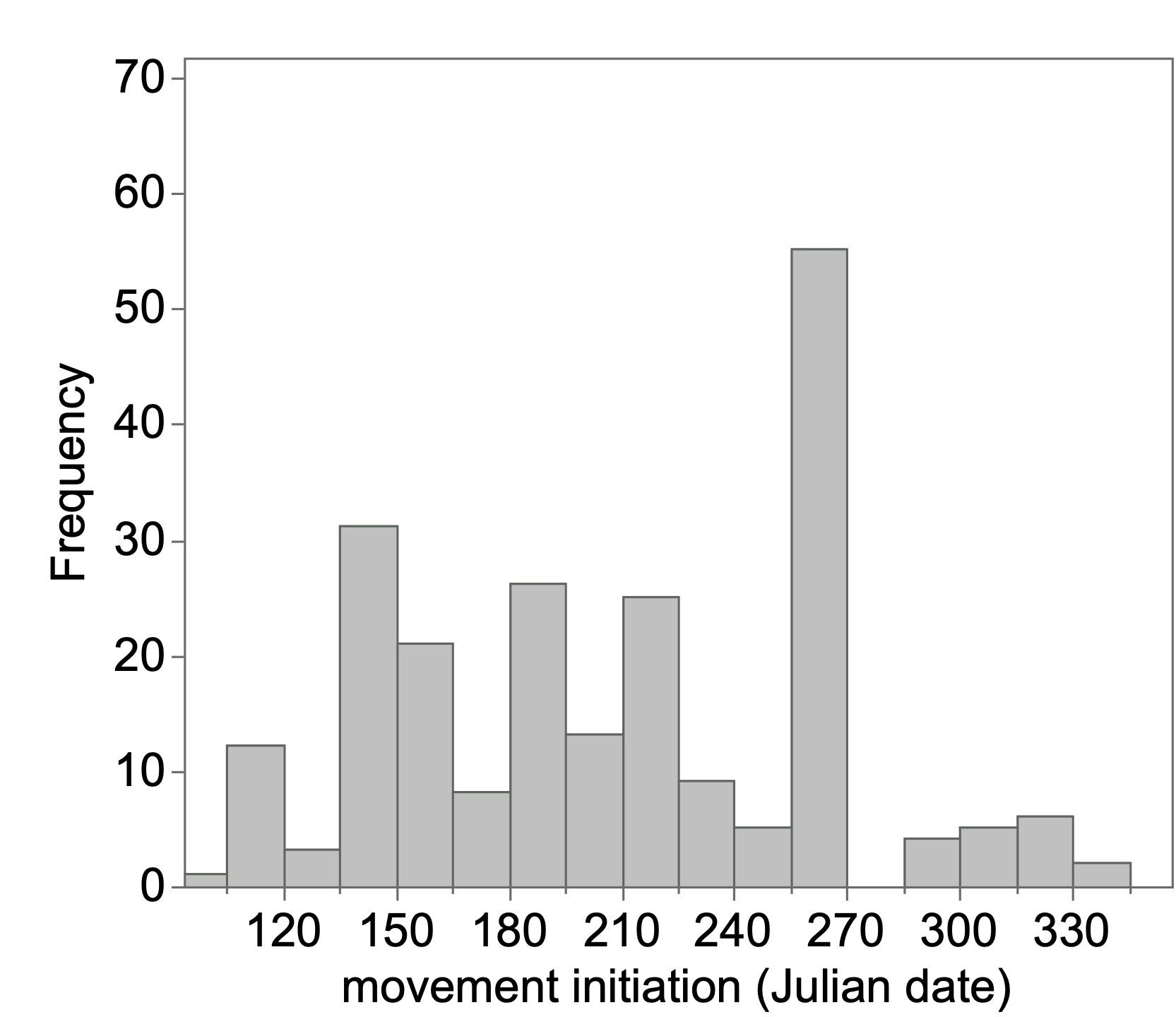
**

**Figure S7:** Julian dates of Grass Pickerel movement initiation in Beaver Creek from 2009 to 2013 (*n* = 171).


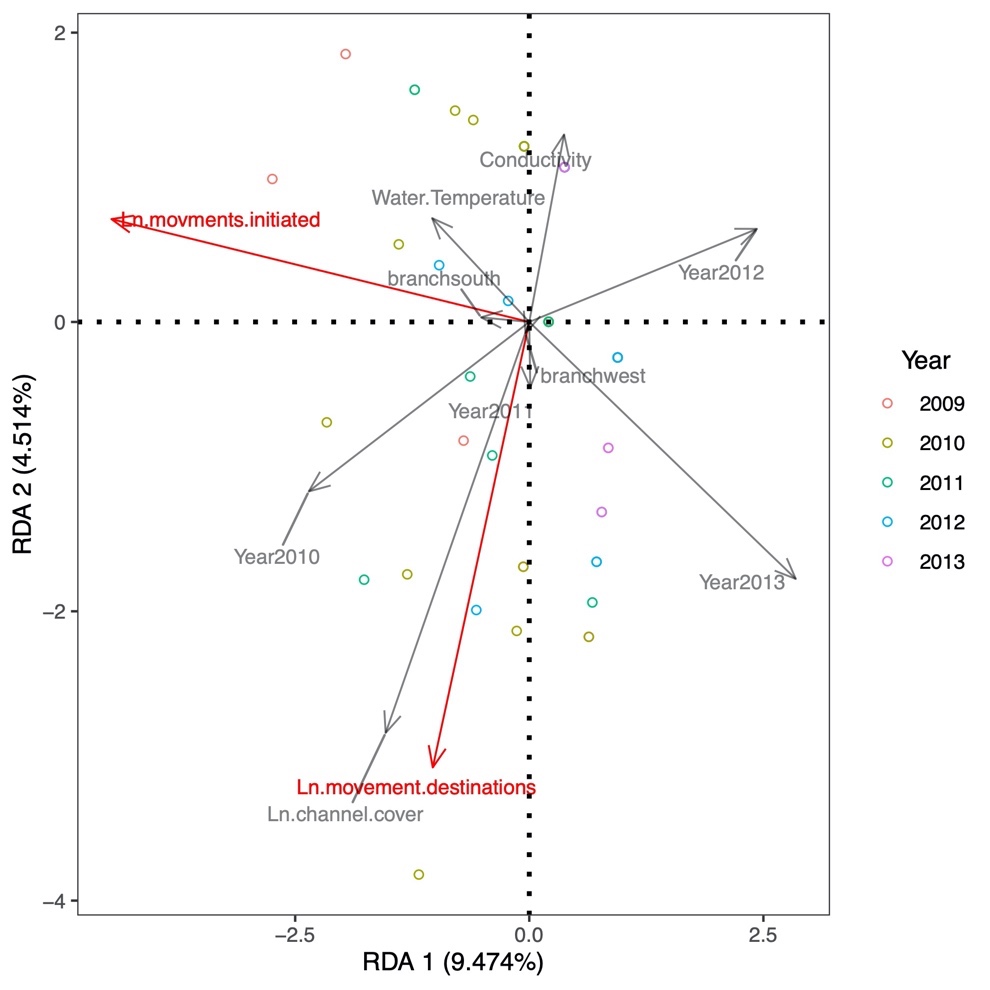


**Figure S8:** Redundancy analysis of Grass Pickerel movements (emigration and immigrations) and habitat variables at surveyed sites in Beaver Creek, Ontario, 2009-2013. Automated recording of the site emigrations and immigrations were summed for two weeks before and after a site observation where habitat variables (vegetation cover, conductivity, water temperature) were measured (*n* = 65). Number of emigrations, immigrations, and channel vegetation cover were transformed to meet assumptions of normality. Observations with zero emigrations, immigrations, or outlier values were removed prior to RDA analysis. The outlier scoring negative RDA2 scores was validated as an aggregate movement where several Grass Pickerel moved to a site within a few days (Figure S5).


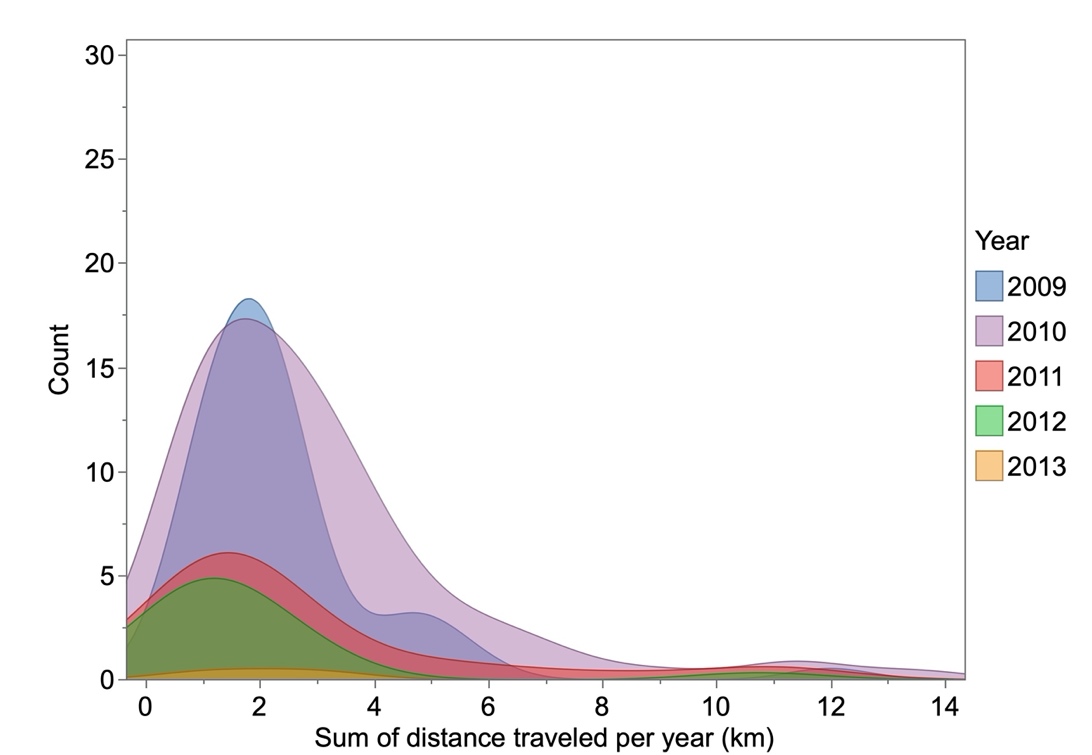


**Figure S9:** Kernel density plots of total annual distance moved by individual Grass Pickerel grouped by year in Beaver Creek from 2009 to 2013. Each curve is a kernel density plot for all movements made by Grass Pickerel for a single year. For Grass Pickerel that made more than one movement in a given year, the distances of all movements during that year were summed. Long-distance movements (> 10 km) occurred in 2009, 2010, 2011, and 2012.
